# Genome-Wide Characterization of the Heat Shock Transcription Factor Gene Family in *Betula platyphylla* Reveals Promising Candidates for Heat Tolerance

**DOI:** 10.3390/ijms26010172

**Published:** 2024-12-28

**Authors:** Shengzhou Guo, Hao Chen, Hongwei Wu, Zuyuan Xu, Hao Yang, Qinmin Lin, Hanyu Feng, Zilu Zeng, Sanjiao Wang, Haolin Liu, Xiaomin Liu, Shijiang Cao, Kang Wang

**Affiliations:** 1 College of Forestry, Fujian Agriculture and Forestry University, Fuzhou 350002, China; gsz19559162600@126.com (S.G.); 18143582739@163.com (Z.X.); m15803366858@163.com (H.Y.); 15879367967@163.com (Z.Z.); 19559168698@163.com (H.L.); 2 College of Computer Science, Fujian Agriculture and Forestry University, Fuzhou 350002, China; chenhaox@outlook.com; 3 Fujian Provincial Key Laboratory of Haixia Applied Plant Systems Biology, Institute of Science and Technology, College of Forestry, Haixia Fujian Agriculture and Forestry University, Fuzhou 350002, China; 19011490579@163.com; 4 College of Life Sciences, Fujian Agriculture and Forestry University, Fuzhou 350002, China; 18760030926@163.com; 5 College of Jixian Honors, Zhejiang Agriculture and Forestry University, Hangzhou 311300, China; fenghanyu042@163.com; 6 State Key Laboratory of Tree Genetics and Breeding, College of Biological Sciences and Technology, Beijing Forestry University, Beijing 100083, China; m17835729632@163.com (S.W.); liuxiaomin@bjfu.edu.com (X.L.)

**Keywords:** *Betula platyphylla*, heat stress transcription factor, subcellular localization, high-temperature stress, gene expression

## Abstract

Heat stress transcription factors (HSFs) play a critical role in orchestrating cellular responses to elevated temperatures and various stress conditions. While extensively studied in model plants, the *HSF* gene family in *Betula platyphylla* remains unexplored, despite the availability of its sequenced genome. In this study, we employed bioinformatics approaches to identify 21 *BpHSF* genes within the *Betula platyphylla* genome, revealing their uneven distribution across chromosomes. These genes were categorized into three subfamilies: A, B, and C. Each was characterized by conserved protein motifs and gene structures, with notable divergence observed between subfamilies. Collinearity analysis suggested that segmental duplication events have driven the evolutionary expansion of the *BpHSF* gene family. Promoter region analysis identified an array of cis-acting elements linked to growth, development, hormonal regulation, and stress responses. Subcellular localization experiments confirmed the nuclear localization of *BpHSFA2a*, *BpHSFB1a*, and *BpHSFC1a*, consistent with in silico predictions. RNA-seq and RT-qPCR analyses revealed tissue-specific expression patterns of *BpHSF* genes and their dynamic responses to heat stress, with qPCR validation highlighting a significant upregulation of *BpHSFA2a* under high-temperature conditions. In summary, this study provided a comprehensive characterization of the *HSF* gene family in *Betula platyphylla*, laying a solid foundation for future functional studies. Particularly, *BpHSFA2a* emerges as a promising candidate gene for enhancing heat tolerance in *Betula platyphylla*, warranting further detailed investigation.

## 1. Introduction

Plants often face a variety of abiotic stresses during growth, especially high-temperature stress, which negatively affect plant growth, organ development, yield, and overall quality. During the course of evolution, plants have gradually constructed a complex transcriptional regulatory system consisting of transcription factors (TFs) and other regulatory elements to cope with external challenges [1]. Transcription factors play a pivotal role in managing both abiotic and biotic stressors, including families such as NAC [2], TCP [3], Dof [4], GATA [5], bHLH [6], among others. Notably, HSF has been recognized as a key regulator in stress responses. Among various abiotic stressors, high-temperature stress (HS) stands out due to its considerable adverse impact on plants [7,8,9]. Heat stress transcription factors represent a distinct class that becomes activated when plants endure increased temperatures. These activated factors can attach to specific DNA sequences known as Heat Shock Response Elements (HSEs), which triggers the transcription of heat shock protein genes, aiding in the protection of plant cells against environmental stressors [10].

**Figure 1:**
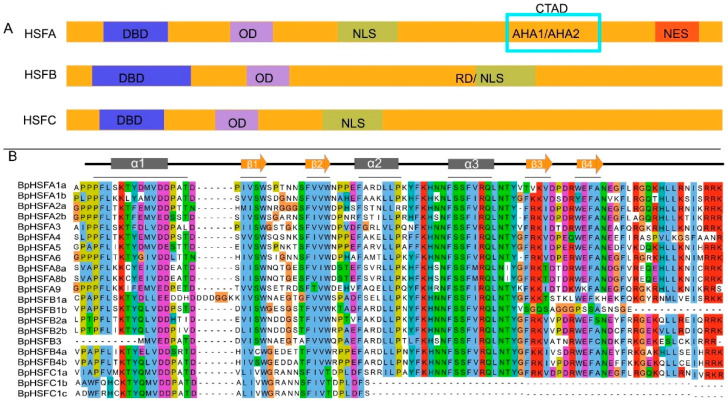
BpHSF protein structural domains and multiple sequence comparison. (**A**) BpHSF protein structural domains, different colored rectangles represent different structural domains (refer to Scharf et al. [18]) (**B**) DBD conserved structural domain of BpHSF. Differences in colour usually indicate regions of amino acids in the amino acid sequence with similar properties or functions.

Similar to many transcription factors, the HSF family exhibits a modular architecture. As illustrated in Figure 1A, the five key structural domains of HSFs include the following: the DNA-binding domain (DBD), the oligomerization domain (OD), the nuclear export signal (NES), the nuclear localization signal (NLS), and the C-terminal transcriptional activation domain (CTAD) [11]. The DBD, located near the N-terminus, is the most conserved region among HSFs and consists of approximately 100 amino acids, which form a hydrophobic core through three α-helices and four β-strands. This domain contains a conserved Helix-Turn-Helix (HTH) motif, a hallmark of catabolic activating proteins, which enables HSFs to bind specifically to Heat Shock Elements (HSEs: 5′-AGAAAnnTTCT-3′), thus modulating the expression of heat stress-responsive genes [12]. The OD domain, present in all HSFs, plays a critical role in oligomerization. It contains two hydrophobic heptapeptide repeat regions, HR-A and HR-B, each forming a characteristic leucine zipper structure. Under heat stress, HSFs oligomerize into homotrimers via this helical structure, facilitating their binding to HSEs and regulating heat stress responses [13]. The OD structure is also pivotal in classifying HSF family members [14]. Based on sequence homology and structural features of the OD, plant HSFs are categorized into three evolutionarily conserved classes: A, B, and C. Classes A and C feature a compact HR-A/B region, whereas class B HSFs exhibit a similar region but lack the amino acid insertions between HR-A and HR-B present in classes A and C—21 and 7 amino acids, respectively [15]. Most HSFs also contain both a nuclear localization signal (NLS) and a nuclear export signal (NES) at the C-terminus. The NLS, adjacent to the C-terminus of the OD, is enriched in basic amino acids such as lysine and arginine, facilitating nuclear entry. Some HSFs additionally possess a leucine-rich NES, which mediates protein export from the nucleus. Together, the NLS and NES work synergistically to regulate the subcellular distribution of HSFs between the nucleus and cytoplasm [16]. The CTAD, located at the C-terminus, is the least conserved region of HSFs and contains a short AHA motif composed of aromatic, hydrophobic, and acidic amino acids [11]. Although less conserved, the CTAD plays a crucial role in transcriptional activation. The AHA motif specifically enables class A HSFs to function as transcriptional activators, while class B and C HSFs, which lack this motif, generally do not exhibit transcriptional activation activity [17].

The Heat Shock Factor (HSF) family of transcription factors consists of highly conserved genes found across both eukaryotes and prokaryotes. The first *HSF* gene was identified and cloned in *Saccharomyces cerevisiae* [18], followed by the discovery of the first plant HSF in tomatoes [19]. With the advent of high-throughput sequencing technologies, an increasing number of *HSF* genes from diverse species have been characterized, including *Arabidopsis thaliana* [20], rice [21], and poplar [22]. These studies have revealed that *HSF* genes play critical roles in regulating plant growth, development, and responses to environmental stress [23]. High temperature is one of the most common abiotic stresses and a major focus of HSF-related research in stress physiology. The HSFA subfamily plays a central role in the heat stress response [24]. The tomato was the first plant to undergo detailed research on heat stress transcription factors, and overexpression of *SlHsfA1* resulted in significantly improved heat tolerance compared to *SlHsfA1*-silenced plants. Similarly, *SlHsfB1* has been shown to enhance heat resistance in tomatoes [25]. In *Arabidopsis thaliana*, *HSFA6b* not only contributes to the heat stress response but also functions as a positive regulator of ABA signaling, improving salt and drought tolerance [26]. Overexpression of *HsfA2* in *Arabidopsis* confers enhanced tolerance to multiple environmental stresses, while knockdown of the gene reduces both basal and acquired thermotolerance as well as tolerance to oxidative stress [27]. In *Capsicum annuum*, the overexpression of *CaHsfA2* [28], as well as the *GmHsf-34* gene in soybean [29], significantly increased tolerance to heat stress in *Arabidopsis thaliana*. Furthermore, heterologous expression of *CsHsfA2* in tea plants enhanced the heat tolerance of transgenic yeast [30]. Recent studies have shown that transient expression of 35S: Gooseberry *LcHsfA2a* in seedlings resulted in significantly lower levels of hydrogen peroxide (H_2_O_2_) and enhanced heat tolerance following heat stress [31]. These studies highlight the important role of HSFs in the plant response to heat stress.

*B. platyphylla* is a deciduous tree belonging to the genus *Betula* of the *B. platyphylla* family and is known for its environmental adaptability. Its wood is highly valued in the production of plywood, furniture, and high-quality paper [32]. It has been found that *B. platyphylla* may face even greater limitations on its growth and development if the current trend of global warming continues or intensifies [33]. Given these challenges, exploring the heat tolerance mechanisms of *B. platyphylla* and enhancing its heat resilience through molecular breeding techniques has become a critical area of research. The recently published genome sequence of *B. platyphylla* provides a solid foundation for studying the *HSF* gene family [34]. Therefore, it is significant for us to study the *HSF* gene family in *B. platyphylla*.

In this study, we systematically identified 21 *HSF* genes in *B. platyphylla* and performed a comprehensive analysis of their gene family using bioinformatics methods. We further explored the heat stress response of *BpHSF* genes, identifying a set of candidate genes with potential roles in heat stress resistance. These findings are crucial for pinpointing key regulators of stress tolerance in *B. platyphylla*. Our results contribute to a deeper understanding of the evolutionary relationships and functional characteristics of the *B. platyphylla HSF* gene family, offering valuable insights for future research on the role of HSF proteins in stress responses and stress-resistant breeding programs.

## 2. Results

### 2.1. Identification and Physicochemical Characterization of the BpHSF Gene Family

After removing redundant sequences, we identified 21 *HSF* genes from the *B. platyphylla* genome using bioinformatics methods. The genes were named *BpHSFA1a* to *BpHSFC1c* based on their phylogenetic relationships with known *Arabidopsis thaliana HSFs*. Detailed information, including the physicochemical properties of the 21 BpHSF proteins, is presented in Table 1. The lengths of the BpHSF proteins ranged from 117 amino acids (*BpHSFC1c*) to 577 amino acids (*BpHSFA3*), with molecular weights varying from 18.69 kDa (*BpHSFC1c*) to 64.05 kDa (*BpHSFA3*). The theoretical isoelectric points (pI) spanned from 4.34 (*BpHSFC1c*) to 8.78 (*BpHSFB1a*). Notably, except for *BpHSFB1a*, *BpHSFB4a*, *BpHSFB4b*, and *BpHSFB2c*, all BpHSF proteins were classified as acidic (pI < 7). The average hydrophilicity values of the BpHSF proteins were negative, indicating a predominantly hydrophilic nature. Additionally, the average instability index of these proteins was calculated to be 55 (above the threshold of 40), suggesting they are likely to be unstable. Subcellular localization predictions revealed that all BpHSF proteins are localized within the nucleus.

### 2.2. BpHSF Protein Multiple Sequence Comparison

The *HSF* gene family protein sequences exhibited five characteristic conserved domains arranged sequentially from the N-terminus to the C-terminus: DBD, OD, NLS, AHA, and NES. Evidently, the DNA-binding domain (DBD) remains the most conserved, with all DBD regions sharing a secondary structure comprising three α-helices and four β-sheets. Variations in insertions or deletions are observed across these sequences. For instance, an 8-amino acid insertion is present in BpHSFB1a between α1 and β1 (Figure 1B). Additionally, substantial amino acid deletions were identified in BpHSFC1b and BpHSFC1c. Consequently, these deletions may render certain biological functions inactive in *B. platyphylla* species.

### 2.3. Chromosomal Localization of BpHSF Genes

**Figure 2:**
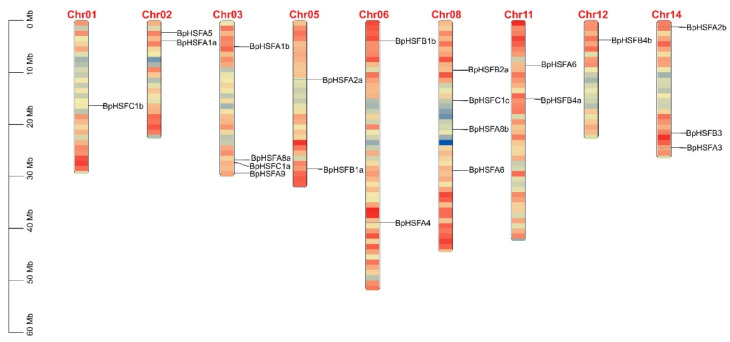
Chromosomal localization of the *BpHSF* gene. *B. platyphylla* has 14 chromosomes. Scale bars are in Mb, and chromosome numbers are shown above the corresponding chromosomes. Chr: Chromosome.

Chromosomal localization analysis revealed that the 21 *BpHSF* genes exhibit distinct distribution patterns across 14 chromosomes, with no *BpHSF* genes detected on chromosomes 4, 7, 9, and 10 (Figure 2). The gene counts vary among chromosomes, with chromosomes 3 and 8 containing the highest number of *BpHSF* genes, each hosting four genes. Specifically, *BpHSFA9*, *BpHSFA1b*, *BpHSFA8a*, and *BpHSFC1* were located on chromosome 3, while *BpHSFA8b*, *BpHSFA2b*, *BpHSFB2a*, and *BpHSFC1c* were mapped to chromosome 8. In contrast, chromosomes 1 and 12 harbored only a single *BpHSF* gene, representing the lowest gene count observed.

### 2.4. BpHSF Evolutionary Tree Analysis

**Figure 3:**
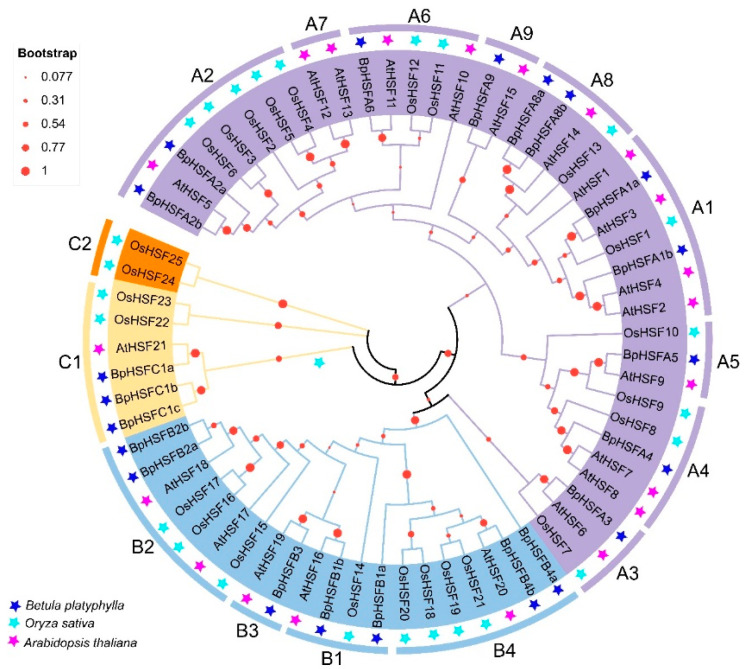
Phylogenetic analysis of *BpHSF* genes was conducted using HSF sequences from *B. platyphylla* (*BpHSF*), *Arabidopsis thaliana* (At), and *Oryza sativa* (Os). As a result, different HSF classes are distinguished by pentagons in various colors, each representing a distinct class.

*HSF* genes from *B. platyphylla*, *Oryza sativa*, and *Arabidopsis thaliana* were compared and analyzed phylogenetically using neighbor-joining phylogenetic trees (Figure 3). This analysis included 67 protein sequences: 21 from *B. platyphylla*, 25 from *Oryza sativa*, and 21 from *Arabidopsis thaliana*. The results classified *BpHSF* genes into three primary groups, corresponding to three subfamilies (A, B, and C) based on the classification systems of model plants *Arabidopsis thaliana* and tomato HSF protein families (Table S1). Subfamily A was further divided into nine subgroups (A1~A9), subfamily B into four (B1~B4), while subfamily C included C1 and C2. In *B. platyphylla*, subfamily A contained eleven *BpHSF* members, subfamily B contained seven, and subfamily C contained three. The distribution of genes across each subgroup varied, with no members of subfamily A7 detected in *B. platyphylla*. Additionally, the C2 subgroup appears unique to the *OsHSF* gene family in *Oryza sativa*.

### 2.5. Protein Motifs and Gene Structure of BpHSF Genes

**Figure 4:**
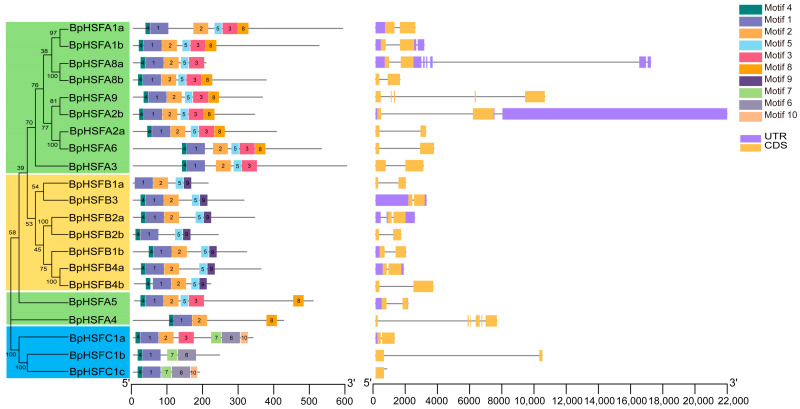
*BpHSF* gene structure and conserved motifs. (**A**) Evolutionary analysis among *BpHSF* genes, subfamily A is indicated in green, subfamily B in yellow, and subfamily C in blue; (**B**) BpHSF protein conserved motifs, indicated by different colored rectangles; (**C**) *BpHSF*gene structure, with orange rectangles indicating CDS (coding sequence), purple rectangles indicating UTRs (untranslated regions), and black lines indicating introns.

Using the MEME tool, we identified 10 conserved motifs within the BpHSF family (Figure 4B), with detailed sequence information provided in Table S2 and Figure S1. The majority of these motifs spanned approximately 50 amino acids, with BpHSFA3 exhibiting the longest sequence and *BpHSFC1c* the shortest. Phylogenetic and gene structure analyses (Figure 4A,C) revealed that members within the same subfamily share similar motifs, while distinct motifs were observed across different subfamilies, suggesting both structural and functional conservation. Within subfamily A, motif patterns were largely consistent, with the exception of *BpHSFA8a*, which lacks motif 8. In subfamily B, *BpHSFA5* and *BpHSFA4* harbor motif 8, while the remaining members exhibit a uniform motif structure. All BpHSF family members contain motifs 1 and 4, and all, except for *BpHSFC1b* and *BpHSFC1c*, also feature motif 2. Interestingly, motifs 10 and 6 are exclusive to subfamily C, while motif 9, with an identical amino acid sequence, is confined to subfamily B.

Exon–intron structure analysis of the 21 *BpHSF* genes revealed that these genes contain between 1–6 exons and 1–5 introns, with the exception of *BpHSFC1c* in subfamily C, which lacks intron. The untranslated region (UTR) structure of the *BpHSF* genes was incomplete: six *BpHSF* genes possess both 5′ UTR and 3′ UTR sequences, while four genes (*BpHSFA9*, *BpHSFB1a*, *BpHSFB4a*, and *BpHSFC1a*) contain only a 5′ UTR. While, 11 genes lack UTR sequences altogether.

### 2.6. Covariance Analysis of BpHSF

**Figure 5:**
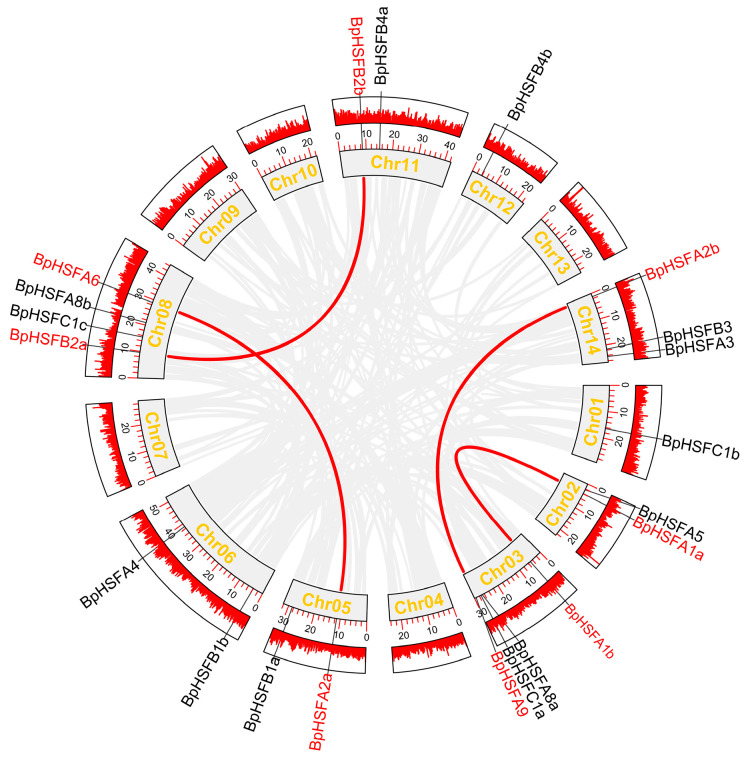
Intraspecies covariance analysis of *BpHSF* genes. The red line in the outer ring represents the gene density per chromosome. Colinear genes are indicated by light gray lines; *BpHSF* gene pairs are shown as red lines. The red circles on the outside represent gene density.

We conducted a gene duplication analysis using MCScanX combined with TBtools-II. The analysis identified one tandem duplication pair (*BpHSFA8a* and *BpHSFC1a*) on chromosome 3 in the *B. platyphylla* genome (Figure 5). Additionally, four segmental duplication events were observed, involving the gene pairs *BpHSFA1a*/*BpHSFA1b*, *BpHSFA6*/*BpHSFA2a*, *BpHSFA2b*/*BpHSFA9*, and *BpHSFB2a*/*BpHSFB2b*. These findings suggest that gene duplication events have been crucial to the evolution and expansion of the *BpHSF* family, with segmental duplications particularly contributing to diversity and functional adaptation within the family.

**Figure 6:**
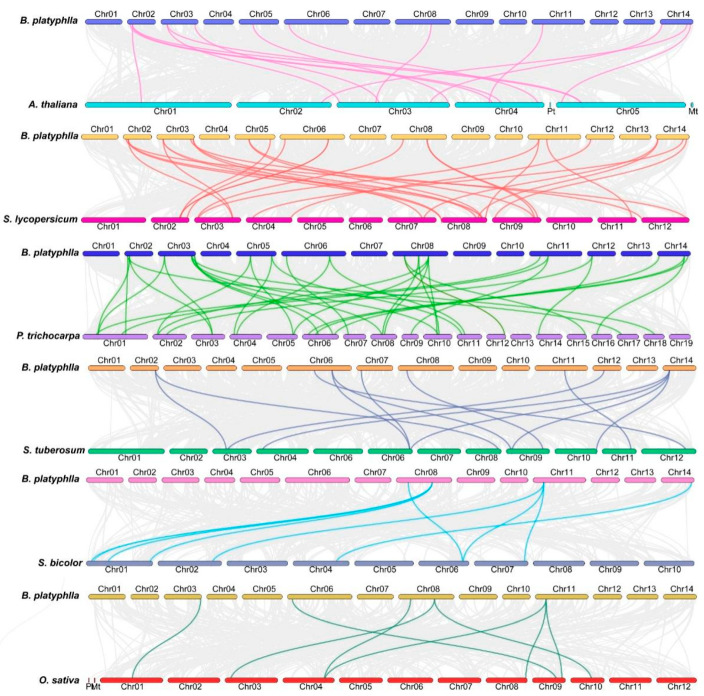
Analysis of *BpHSF* gene colinearity with six representative plants. In the figure, gray lines indicate regions of colinearity within the genomes of *B. platyphylla* and other plants, while other colored lines highlight the colinear *HSF* gene pair.

To explore the evolutionary trajectory of the *BpHSF* genome, we conducted a comparative genomic analysis involving six representative species: four dicotyledons (*Arabidopsis thaliana*, potato, tomato, and poplar) and two monocotyledons (*sorghum* and *Oryza sativa*) (Figure 6). This analysis uncovered syntenic relationships between *B. platyphylla* and all six species, with 14 syntenic pairs shared with *A. thaliana*, 32 with *Poplar*, 22 with tomato, 19 with potato, 11 with pineapple, and 8 with sorghum. Notably, *B. platyphylla* exhibited the highest number of collinear gene pairs with poplar (32 pairs), suggesting a significant homologous relationship and extensive gene exchange during their evolutionary history. Although synteny was observed across all species, a distinct pattern emerged, with stronger synteny observed in dicotyledons compared to monocotyledons, underscoring closer evolutionary affinities among dicot species.

### 2.7. Analysis of BpHSF Promoter Cis-Acting Elements

**Figure 7:**
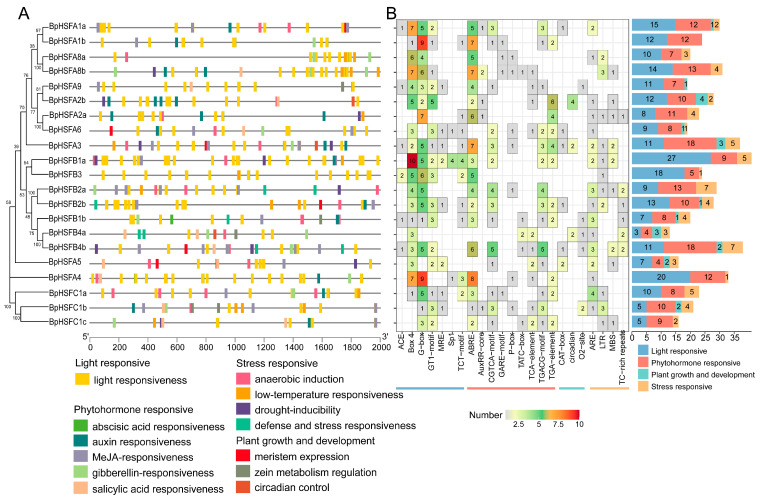
Analysis of cis-acting regulatory elements (CREs) in the *BpHSF* gene family. (**A**) Positional distribution of various CRE types within the promoter regions, with distinct colored boxes representing different CRE types, some of which may overlap. (**B**) Number of cis-acting elements in the promoter regions of *BpHSF* genes, displayed in a heatmap where each cell indicates the number of specific CREs; cells in white denote the absence of corresponding elements.

Transcription factors are essential in mediating plant responses to biotic and abiotic stresses by regulating various cis-regulatory elements within gene promoter sequences. To explore the potential biological functions and regulatory network of *BpHSF*, we analyzed cis-regulatory elements within the promoter regions of *BpHSF* genes (spanning 2000 bp upstream of the start codon). This analysis identified a total of 533 cis-regulatory elements (Table S3), categorized into four groups: light response, stress response, hormone response, and growth and development response (Figure 7A). Light-responsive elements were the most prevalent, comprising 237 elements and accounting for 44.5% of the total, followed by hormone-responsive elements (208, or 39%). Growth and development-responsive elements were the least frequent, potentially indicating a more limited direct role for the *BpHSF* gene family in plant growth and development.

A diverse array of cis-regulatory elements was identified within the *BpHSF* genes, including light-responsive G-boxes, abscisic acid-responsive elements (ABREs), anaerobic-inducible elements (AREs), drought-responsive MYB-binding sites (MBSs), and low-temperature-responsive elements (LTRs), suggesting a pivotal role for the *BpHSF* gene family in mediating environmental stress responses (Figure 7B). Notably, no cis-elements directly linked to high-temperature responses were detected. Among the 21 *BpHSF* genes, four (*BpHSFA1a*, *BpHSFA3*, *BpHSFB1a*, and *BpHSFB4b*) harbored more than 30 cis-regulatory elements each. Stress-related elements were particularly prevalent in BpHSFB2a, BpHSFB4b, BpHSFA3, BpHSFB1a, and BpHSFA2, highlighting their likely involvement in plant responses to environmental stressors, including heat, salinity, and drought.

### 2.8. Secondary and Tertiary Structure Prediction and Subcellular Localization of the BpHSF Protein

**Figure 8:**
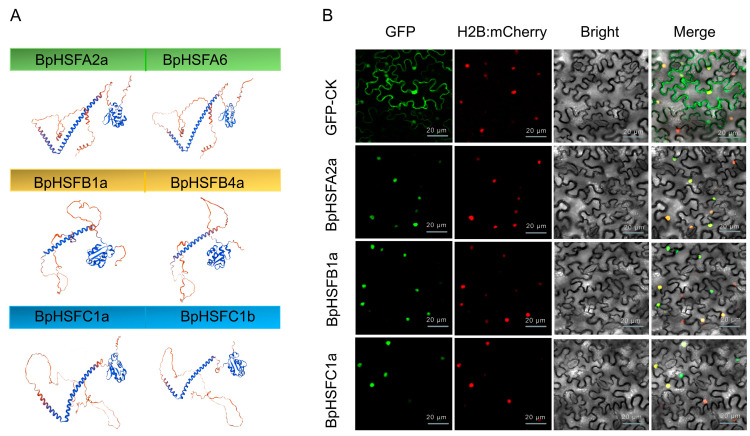
Secondary and tertiary structure and subcellular localization of BpHSF proteins. (**A**) Predicted 3D structure of the BpHSF protein sequence, with α-helical domains indicated in blue and β-turn domains in dark red. (**B**) Subcellular localization of BpHSF proteins. Nuclei were visualized using a co-transformed mCherry-labeled nuclear marker (H2B-mCherry). Scale bar = 20 μm.

We further investigated the protein structures of *BpHSF* genes. The results showed that all of these proteins include α-helix, β-turn, irregular coil, and extended chain, but the proportion of the secondary structure elements is variable, specifically, the proportion of irregular coil is the highest, varying from 31.12% to 59.1%, followed by α-helix (13.45% to 56.40%), and β-turn is the lowest, ranging from 1.86% to 10.73%. α-helix accounts for a high percentage, which can predict that the protein has a high stability (Figure S2). Conserved structural domains of BpHSF proteins were visualized, and six 3D structural models of BpHSF proteins were predicted (Table S4). The 3D structural models clearly showed the α-helical and β-folded structures of the DBD structural domains (Figure 8A). Further analysis revealed that BpHSF proteins of the same subfamily have similar three-dimensional structures. The tobacco transient expression analysis revealed that BpHSFA2a, BpHSFB1a, and BpHSFC1a were localized within the nucleus (Figure 8B).

### 2.9. Expression of BpHSF Gene in Different Tissues and Its Response to High-Temperature Stress

**Figure 9:**
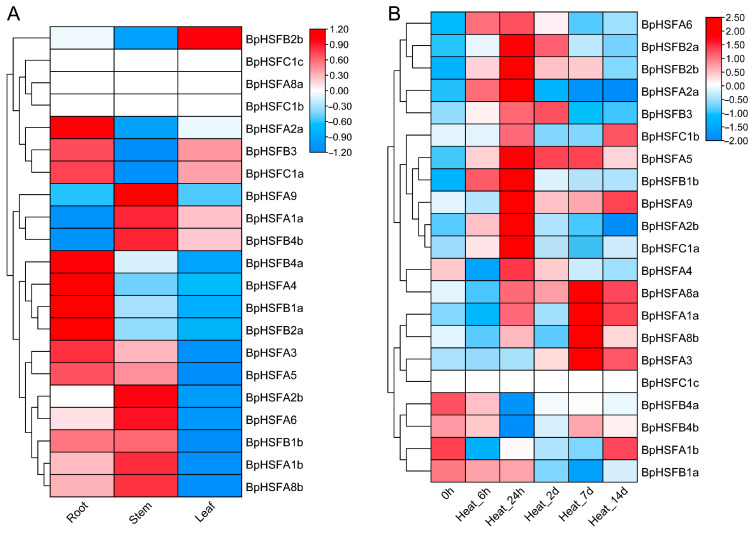
Transcriptome-based expression profiling of *BpHSF* genes. (**A**) Tissue-specific expression patterns of *BpHSF* genes across three different tissues: roots, stems, and leaves. (**B**) Differential expression patterns of *BpHSF* genes under heat stress conditions. The color bars represent normalized expression levels (log2-transformed fold change), with red indicating upregulated genes, blue indicating downregulated genes, and white indicating no expression.

The expression profiles of the 21 *BpHSF* genes were evaluated using the FPKM method, based on transcriptome data from three distinct tissues of *B. platyphylla*: roots, stems, and leaves (Table S5, Figure 9A). The analysis revealed substantial variation in the expression levels of *BpHSF* genes across these tissues, suggesting a tissue-specific regulatory mechanism. Notably, three genes—*BpHSFA8a*, *BpHSFC1b*, and *BpHSFC1c*—exhibited persistently low expression across all tissues, indicating that their roles may be limited or non-essential in these physiological contexts. In contrast, other *BpHSF* genes displayed distinct tissue-specific expression patterns. For instance, *BpHSFB1b* and *BpHSFB2a* were highly expressed in leaves, while *BpHSFA2a*, *BpHSFA4*, *BpHSFA5*, and *BpHSFB1a* were predominantly expressed in roots. Additionally, *BpHSFA9*, *BpHSFA2b*, and *BpHSFA6* showed elevated expression in stems. These divergent expression profiles suggest that *BpHSF* genes play critical roles in the growth, development, and adaptive responses of *B. platyphylla*.

To further elucidate the role of *BpHSF* genes in the heat stress response, we analyzed their expression profiles using previously acquired transcriptome data (Table S5, Figure 9B). After 24 h of heat stress, a subset of *BpHSF* genes, including *BpHSFB2a*, *BpHSFB2b*, *BpHSFA2a*, *BpHSFA5*, *BpHSFB1b*, *BpHSFA9*, *BpHSFA2b*, and *BpHSFC1a*, were significantly upregulated, underscoring their critical involvement in the early heat stress response of *B. platyphylla*. In contrast, *BpHSFC1c* exhibited no detectable expression, suggesting either an absence of heat responsiveness or its lack of expression in *B. platyphylla* leaves. Prolonged heat exposure resulted in a notable downregulation of genes that were initially upregulated, such as *BpHSFA2a*, *BpHSFA2b*, *BpHSFB1b*, and *BpHSFC1a*, potentially reflecting an adaptive energy-conserving mechanism. Meanwhile, genes such as *BpHSFA8a*, *BpHSFA1a*, *BpHSFA8b*, and *BpHSFA3* showed significant upregulation after seven days of heat stress, implying their involvement in the late-phase heat stress response.

### 2.10. BpHSF Expression Validation Through qRT-PCR

**Figure 10:**
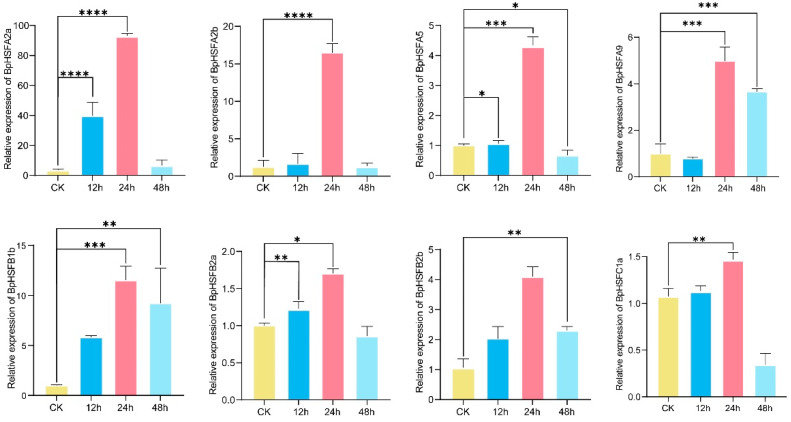
qRT-PCR analysis of selected highly responsive *BpHSF* genes in leaf tissues under high-temperature stress. Asterisks above the bars indicate statistically significant differences between the stress-treated samples and their corresponding controls (* *p* < 0.05, ** *p* < 0.01, *** *p* < 0.0005, **** *p* < 0.0001).

Eight heat stress transcription factor genes (*BpHSFB2a*, *BpHSFB2b*, *BpHSFA2a*, *BpHSFA5*, *BpHSFB1b*, *BpHSFA9*, *BpHSFA2b*, and *BpHSFC1a*), identified as highly expressed under high-temperature conditions through transcriptome analysis, were further validated using qRT-PCR. The results (Figure 10) revealed that all eight genes reached peak expression levels after 24 h of heat treatment, followed by varying degrees of decline as stress exposure elevated. Particularly, *BpHSFA2a* and *BpHSFA2b* showed significant upregulation at 24 h. *BpHSFA2a*, in particular, exhibited an exceptional increase, being unregulated 80–100-fold compared to the control, but its expression rapidly returned to baseline levels after 48 h, indicating a crucial role in the early stages of heat stress response. Further analysis indicated that members of the A subfamily (*BpHSFA2a*, *BpHSFA2b*, *BpHSFA5*, and *BpHSFA9*) had higher expression levels at 24 h compared to those of the B and C subfamilies, suggesting an important role for A subfamily genes in the heat stress response. Conversely, *BpHSFC1a*, a member of subfamily C, exhibited the lowest expression level at 24 h, being only 1–1.5-fold that of the control, implying a limited role in high-temperature response.

## 3. Discussion

HSFs are a class of transcription factors that play crucial roles in mediating plant responses to heat and various environmental stresses. The HSF gene family has been identified and analyzed in numerous terrestrial plant species. However, the number and composition of HSF gene families vary among species, likely reflecting differences in ecological adaptation, evolutionary history, and environmental stress response mechanisms. HSF gene families have been studied in different plants, e.g., eggplant contains 24 SmHSFs [35], walnut has 29 JrHSFs [36], kale-type oilseed rape has 64 HSFs [37], while bread wheat has 78 HSFs [38], and wheat has 82 TaHSFs [39]. Despite these studies, the identification and characterization of the HSF gene family in *B. platyphylla* have not yet been reported. With the release of the *B. platyphylla* genome, we have now identified 21 *BpHSF* genes, which are unevenly distributed across 14 chromosomes. Interestingly, chromosome length did not necessarily correlate with the number of HSF genes. In angiosperms, the HSF gene family is divided into three subfamilies: A, B, and C. Subfamily A includes A1–A9, subfamily B includes B1–B5, and subfamily C contains C1. Based on gene structure and phylogenetic relationships, we classified the 21 *BpHSF* genes into three subfamilies: A, B, and C. Specifically, subfamily A contains 11 genes, subfamily B contains 7 genes, and subfamily C contains 3 genes. Although the total number of HSF genes in *B. platyphylla* is similar to that of Arabidopsis, differences were observed in the distribution of specific HSF subclasses. Crucially, the number of HSFA1 subclass members is higher in *B. platyphylla*, while no members of the HSFA7 subclass were identified. Additionally, there is only one member of the HSFA9 subclass, compared to four in chili peppers [28]. Subfamily C includes three members, of which two belong to class C2, consistent with findings in *Oryza sativa* (OsHSF) [40] and Minnan (PbHSF) [41], both monocotyledonous plants. Interestingly, the HSFB5 subclass was absent in all of the above studies, further supporting the accuracy of our identification of *BpHSF* genes in *B. platyphylla*. Analysis of the physiochemical properties of the proteins encoded by the *BpHSF* genes indicated that all of the proteins are hydrophilic and predominantly localized in the nucleus. This observation is consistent with findings in carnations [42,43] and turnips [44], suggesting a similar pattern of subcellular localization for HSF proteins.

The highly conserved DNA-binding domain (DBD) consists of approximately 100 amino acid residues across various plant species. Strikingly, the DBDs of *BpHSFB1b*, *BpHSFC1a*, and *BpHSFC1b* are shorter compared to other *BpHSFs*, possibly due to the genomic or genetic variation in *B. platyphylla*. Members of the same subfamily within the phylogenetic tree share consistent motif alignments, with motifs 1, 2, and 4 being present in nearly all *BpHSFs*. The similarity in motif composition among members of the same subfamily suggests that these proteins have conserved structural and functional roles, potentially sharing a common ancestor and being genetically more closely related. In plants, introns are crucial in regulating gene expression. While introns themselves do not encode proteins, they contribute to regulating gene expression, generating alternative mRNA isoforms, and influencing gene stability. Therefore, studying the structural features of *BpHSF* genes can provide deeper insights into their functions. Our analysis revealed that nearly all *BpHSF* genes contain between one and five introns, whereas *BpHSFC1c* lacks introns, which might imply a limited role in gene expression regulation. Additionally, *BpHSF* genes exhibit significant variation in the location and length of introns, suggesting functional divergence between subfamilies. In the prediction of three-dimensional structures, BpHSF proteins within the same subfamily manifested highly similar structural characteristics. These findings are consistent with studies of HSF genes in papaya [45] and poplar [46], which also showed similar motif compositions and tertiary structures. Previous studies have reported that most HSFs are localized in the nucleus, such as Rhododendron (*RsHsf15*, *RsHsf16*, and *RsHsf19*) [47], peanut (*AhHsf20*) [48], *Salvia divinorum* (*SmHsf1* and *SmHsf7*) [49], wheat (*TaHsfA2-10*) [50], and buckwheat (*FtHsf18* and *FtHsf19*) [51]. Consistent with these findings, our study demonstrated that *BpHSFA1a*, *BpHSFB1a*, and *BpHSFC1* are all localized in the nucleus.

Studying gene duplication events and synteny relationships provides critical insights into the expansion, functional diversification, and evolutionary history of the *BpHSF* gene family. In the *BpHSF* gene family, only one tandem duplication event was identified, whereas five gene pairs exhibited segmental duplication events. These findings suggest that segmental duplication may have been the primary driver behind the expansion of the *BpHSF* gene family in *B. platyphylla*. A similar pattern has been observed in passion fruit [52] and hawthorn [53]. Covariance analysis among representative species revealed that *B. platyphylla* and Populus shared the highest number of covariant gene pairs (32), indicating frequent gene exchanges or shared genomic structures between these species during evolution. This evolutionary analysis of *BpHSF* genes provides an essential foundation for further functional and evolutionary studies. Cis-regulatory elements (cis-elements) in the promoter regions of gene family members are specific DNA sequences that regulate gene transcription by binding to transcription factors and other regulatory proteins, thereby precisely controlling the level and pattern of gene expression. Promoter analysis revealed that the promoter regions of *BpHSF* genes harbor different numbers and types of cis-elements, suggesting varied transcriptional regulation among these genes. The identified cis-elements include those responsive to stress, light, hormones, and growth and development, indicating the diverse regulatory roles of *BpHSF* genes. Interestingly, no Heat Shock Elements (HSEs) were detected in the *BpHSF* promoter regions, which aligns with findings in cannabis [54] and wild grape [55]. However, the presence of HSEs in the barley *HSF* gene family [56] suggests that the mechanism of *HSF* gene response to heat stress may vary among plant species. It remains unclear whether the expression of the *BpHSF* genes in *B. platyphylla* is directly regulated by heat stress, given the absence of typical HSE elements. Possibly the *BpHSF* genes responded to heat stress indirectly through other cis-elements. Therefore, further experimental studies are needed to verify the regulation of *BpHSF* gene expression under heat stress conditions and to elucidate potential alternative regulatory mechanisms involved in environmental stress responses.

**Figure 11:**
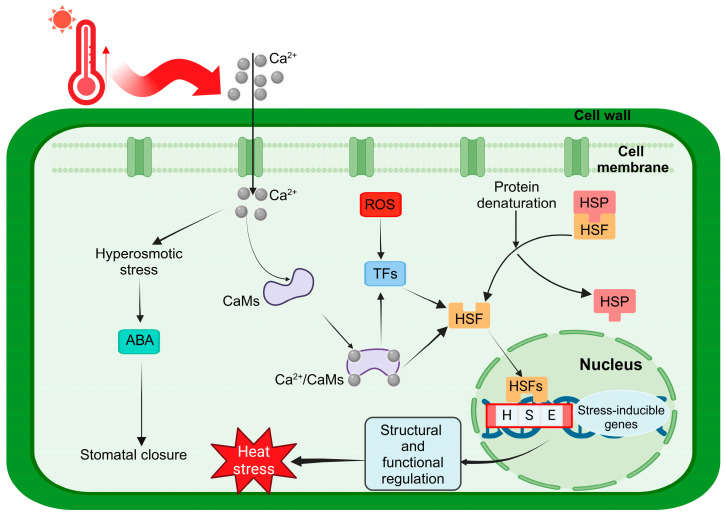
Schematic representation of the mechanism underlying HSF transcription factor response to high temperature (modified from Bakery et al. [70]; Zhang et al. [71]; Guo et al. [72]).The red arrow indicates an increase in temperature. CaM, calmodulin; ROS, reactive oxygen species; TF, transcription factor; HSP, heat shock protein; ABA, abscisic acid; HSF, heat shock transcription factor; HSE, Heat Shock Element.

Through RNA-seq analysis and qPCR validation, we observed that the *BpHSF* genes of *B. platyphylla* reached peak expression after 24 h of high-temperature treatment, with subfamily A genes (e.g., *BpHSFA2a* and *BpHSFA2b*) showing particularly significant responses. Among them, *BpHSFA2a* exhibited the most pronounced response. These findings are consistent with previous studies in *Camellia sinensis* (tea tree) [30], *Triticum aestivum* (wheat) [57], and *Capsicum annuum* (chili pepper) [28], supporting the idea that HsfA subfamily genes play a crucial role in adopting plants to high-temperature stress by rapidly activating heat-responsive genes [36]. In addition, a recent study found that the transcription factor *LpHSFA3*, acting as a positive regulator of *LpHSFA2a*, enhances the heat tolerance of perennial ryegrass [58]. Similarly, *DcaHsfA2a* and *DcaHsfA2b* in carnations have been identified as potential candidates for heat tolerance breeding [42]. Under high-temperature stress, *Zea mays* (maize) genes *ZmHsf-01* (A2), *ZmHsf-03* (B), *ZmHsf-04* (A2), *ZmHsf-23* (A6), *ZmHsf-24* (A9), and *ZmHsf-25* (B) also showed significant upregulation [59]. In soybeans, overexpression of the *GmHsf-34* (A2) gene increased heat tolerance in *Arabidopsis thaliana* [29]. Some studies have suggested *HSFC1* as a key gene for enhancing plant tolerance to heat and fungicidal stress [60,61]. Our findings indicated that the response of *BpHSFC1a* was not significant under high-temperature conditions, which diverges from previous reports. For example, *PeHSF-C1a* in passion fruit has been reported to be involved in the heat stress response [52]. Studies in *Populus* and *Arabidopsis thaliana* [62,63] have shown that *HSF* genes are often activated within 15 to 30 min of high-temperature treatment; similarly, genes such as *LcHsfA2a*, *LcHsfA2b*, and *LcHsfA3* are highly expressed in loblolly pine after just one hour of heat exposure [31]. In contrast, *BpHSF* genes in *B. platyphylla* reached their peak expression after 24 h, suggesting that white birch may employ a unique adaptive mechanism that favors a sustained response to cope with prolonged high-temperature stress. In this study, we found that birch *HSF* genes exhibited diverse expression responses to high-temperature stress, similar to the signaling and biological processes observed in most plants under heat stress. Thus, the *HSF* gene family plays an important role in coordinating plant responses to high-temperature stress, acting as a key component of stress signaling (Figure 11).

Under heat stress conditions, even in the absence of Heat Shock Elements (HSEs), the steady-state levels of *BpHSFA2a*, *BpHSFA2b*, *BpHSFB1b*, and *BpHSFC1a* transcripts were significantly upregulated. Among these, the pronounced expression of *BpHSFA2a* was primarily attributed to post-transcriptional regulatory mechanisms and enhanced transcript stability. Heat stress likely modulates the 5′ or 3′ untranslated regions (UTRs) of target transcripts by inducing the expression and activity of specific RNA-binding proteins (RBPs) or non-coding RNAs (ncRNAs) [64]. These factors act to protect the mRNAs from degradation and extend their half-lives [65]. Additionally, this regulatory mechanism may involve indirect signaling pathways activated by heat stress, such as those mediated by ABA-responsive elements (ABREs) or antioxidant response elements (AREs), which further amplify the expression of associated genes [66,67]. The remarkable upregulation of *BpHSFA2a*, in particular, may result from its transcript’s higher affinity for stability factors induced by heat stress or the intrinsic structural features of its mRNA that confer greater stability [68]. These properties likely provide *BpHSFA2a* with a competitive advantage in expression under heat stress, allowing it to dominate at the steady-state level. The critical role of post-transcriptional mechanisms in regulating heat stress-responsive gene expression is further underscored by the specificity of RNA-binding proteins in recognizing target transcripts and the multi-layered regulatory effects of ncRNAs within gene regulatory networks [69]. This highlights the intricate post-transcriptional control mechanisms that ensure efficient gene expression during heat stress.

When ambient temperatures rise to approximately 40 °C, intra-cellular calcium ion (Ca^2^⁺) levels increase, allowing Ca^2^⁺ to enter the cell and bind to calmodulin (CaM), forming the Ca^2^⁺/CaM complex. This complex activates various downstream responses, including reactive oxygen species (ROS) signaling and the activation of transcription factors (TFs). Under heat stress, protein denaturation leads to the dissociation of HSF from heat shock proteins (HSPs), resulting in HSF activation. Activated HSF binds to Heat Shock Elements (HSEs) in gene promoters, initiating the expression of HSPs. HSPs assist cells in resisting protein denaturation, maintaining protein stability and function, thereby protecting cells from heat-induced damage. The accumulation of HSPs, along with other stress responses (e.g., ABA-induced stomatal closure), collectively enhances plant tolerance to high-temperature stress, promoting structural and functional regulation and improving overall stress resilience.

## 4. Materials and Methods

### 4.1. BpHSF Gene Family Identification and Characterization

The complete genome and gene annotation (gff3) file of *B. platyphylla* were downloaded from Phytozome and analyzed using TBtools-II software (version 2.056) [73]. These sequences were compared using TBtools-II, and a bidirectional BLAST search was performed against the full gene sequence database of *B. platyphylla*, applying a filtering threshold of E ≤ 10⁻^5^. The resulting sequences were considered as potential HSF family members in *B. platyphylla*. Simultaneously, the Hidden Markov Model (HMM) file for HSF (Pfam no.PF00447) was obtained from the InterPro (https://www.ebi.ac.uk/interpro, accessed on 6 May 2024) database, and HSF-related sequences were screened using HMMER 3.0 software to identify those containing HSF structural domains within the *B. platyphylla* genome. Protein sequences from *B. platyphylla* were similarly screened for HSF domains using the Hidden Markov Model. Candidate protein sequences from both methods were refined by removing duplicates and incomplete sequences. The remaining sequences were intersected using Venn plots in TBtools-II. Finally, functional structural domains were verified using the SMART online tool (http://smart.embl-heidelberg.de/ (accessed on 12 May 2024)), and sequences with missing or incomplete domains were excluded. As a result, 21 *B. platyphylla* HSF transcription factor family members were identified. The structural and physicochemical properties of the *B. platyphylla* HSF transcription factor proteins were analyzed using the Expasy ProtParam (https://web.expasy.org/protparam/ (accessed on 14 May 2024)) tool, which provided information on the number of amino acids (AAs), molecular formula (MF), molecular weight (MW), theoretical isoelectric point (pI), instability index (II), aliphatic index (AI), and Grand average of hydropathicity (GRAVY). Subcellular localization (SL) prediction of the *B. platyphylla* HSF transcription factor gene family using the online tool WoLF PSORT (https://wolfpsort.hgc.jp/?utm_source=chatgpt.com (accessed on 15 May 2024)).

### 4.2. Multiple Sequence Alignment, Chromosomal Localization, and Phylogeny

CLUSTALW (https://www.genome.jp/tools-bin/clustalw (accessed on 2 June 2024)): we performed multiple sequence alignment on the BpHSF protein and visualized the structural domains of the BpHSF protein sequence using Jalview software (version 2.11.3.0). In order to generate a chromosome map of the HSF gene family in birch, TBtools II software was used to integrate the GFF3 file of birch with the BpHSF gene ID obtained through comparison. CLUSTALW using default parameters (https://www.genome.jp/tools-bin/clustalw (accessed on 3 June 2024)): Multiple sequence alignment was performed on the full-length HSF amino acid sequences of birch, rice, and Arabidopsis. Then, MEGA11 software was used for phylogenetic analysis, and the neighbor connection (NJ) method was applied for 1000 guided replicates. A phylogenetic tree was generated using iTOL visualization (https://itol.embl.de/ (accessed on 12 June 2024)).

### 4.3. BpHSF Conserved Motif Prediction and Gene Structure Analysis

The conserved motifs of gene families were predicted using MEME (http://memesuite.org/tools/meme (accessed on 17 June 2024)) with the motif parameter set to 10, and the MAST XML output file was downloaded for visualization using TBtools-II. The *B. platyphylla HSF* gene sequences and their corresponding mRNA sequences were extracted, and the exon–intron structures were analyzed and visualized using the GSDS tool (http://gsds.cbi.pku.edu.cn/ (accessed on 24 June 2024)) [74].

### 4.4. Analysis of BpHSF Covariance and Cis-Acting Elements

To investigate the evolutionary expansion and selective pressure acting on the *B. platyphylla* HSF gene family, gene covariance analysis was conducted. Tandem duplication events among *B. platyphylla* HSF genes were identified using TBtools-II and MCScanX tools. Subsequently, segmental duplication events and inter-species gene covariance were analyzed using TBtools-II, MCScanX (https://github.com/wyp1125/MCScanX (accessed on 12 July 2024), and BLASTP (https://blast.ncbi.nlm.nih.gov/Blast.cgi?PROGRAM=blastp (accessed on 24 July 2024)).

Promoter regions, defined as 2 kb upstream of the transcription start site of all *BpHSF* genes, were extracted using TBtools-II and submitted to the PlantCARE database (https://bioinformatics.psb.ugent.be/webtools/plantcare/html/ (accessed on 14 July 2024)) for cis-acting element analysis. All cis-elements in the promoter regions were screened, and analyzed for redundancy, and those with significant functional importance were selected and visualized using TBtools-II.

### 4.5. BpHSF Protein Structure Prediction and Subcellular Localization

SOPMA (https://npsa-prabi.ibcp.fr/cgi-bin/npsa_automat.pl?page=npsasopma.html (accessed on 13 August 2024)) is used for protein secondary structure prediction, using default parameters. The SWISS-MODEL database (https://swissmodel.expasy.org/ (accessed on 14 August 2024)) was used to predict the tertiary structure of proteins by homology modeling.

To analyze subcellular localization, transient expression assays were performed in tobacco. Tobacco plants were grown for 4–5 weeks under conditions of 14 h light/10 h dark, 25 °C, and 70% relative humidity. Full-length CDSs of *BpHSFA2a*, *BpHSFB1a*, and *BpHSFC1a* were amplified from *B. platyphylla* leaf cDNA, using primers detailed in Table S6, and cloned into the pCAMBIA2300-eGFP vector (Fenghui Biotechnology, Changsha, China). The resulting expression plasmids were transformed into Agrobacterium tumefaciens strain GV3101, with appropriate antibiotics added during culture. The constructed Agrobacterium was then injected into leaves of *Nicotiana benthamiana*, with the pCAMBIA2300-eGFP empty vector serving as a control. Following injection, tobacco plants were kept in darkness for 12 h before being transferred to normal light conditions. After 40 h, fluorescence in the injected areas was observed using a laser confocal microscope (Olympus FV1200 (Olympus Corporation, Tokyo, Japan)), and images were captured. The imaging data were subsequently processed using Adobe Photoshop 2024.

### 4.6. Plant Materials and Treatments

The experiment was conducted at the field base of Fujian Agriculture and Forestry University. Healthy *B. platyphylla* seeds were first selected, then soaked, and germinated before being sown in 12-hole seedling trays filled with specialized seedling substrate. The seedlings were kept moist by daily moderate watering to maintain optimal substrate moisture for approximately two weeks. The *B. platyphylla* seedlings were subsequently grown in a greenhouse at 25 °C, with a relative humidity of 60–70%, a photoperiod of 16 h light/8 h dark, and regular moderate watering. After 24 weeks, uniformly grown plants were transplanted from seedling trays and acclimated in Hoagland nutrient solution for one week.

High-temperature stress treatment was conducted using a multi-chamber, multi-temperature gradient thermostat incubator (model: MTI-201B/202B, five chambers, 31 L per chamber). Samples of *B. platyphylla* roots, stems, and leaves were collected at room temperature (25 °C). For the high-temperature treatment, the temperature was set to 40 °C, with a humidity of 65% light intensity of 100–110 µmol/(m^2^·s), and sampling time points at 0, 12, 24, and 48 h. *B. platyphylla* leaves collected at 0 h served as the control group. All samples were immediately frozen in liquid nitrogen and stored at −80 °C for further analysis. Each treatment was performed in three biological replicates, with 12 *B. platyphylla* seedlings randomly selected per replicate.

### 4.7. Expression Profiles Based on RNA-Seq Data

Publicly available *Betula platyphylla* RNA-seq raw sequencing data were obtained from the NCBI Sequence Read Archive (SRA), including roots (accession no. SRR8953232), stems (accession no. SRR8953231), leaves (accession no. SRR28840994), and samples subjected to high-temperature conditions (accession no. SRP361895) at time points of 6 h, 24 h, 2 days, 7 days, and 14 days, with 0 h (25 °C) serving as the control. The raw sequencing data were initially filtered using Fastp v0.23.1 [75] with default parameters to obtain clean data for subsequent analysis. The filtered data were then aligned to the *B. platyphylla* genome using Hisat2 v2.1.0 [76]. Transcript quantification for each sample was performed using featureCounts v2.0.3 [77]. Gene expression was calculated as the FPKM value for each *BpHSF* member, log2-transformed, and then visualized as a heatmap using the pheatmap package in R software (version 4.4.2).

### 4.8. RNA Extraction and Quantitative Real-Time PCR Analysis

Total RNA was extracted from each tissue sample using the RNA prep PureTotal RNA Extraction Kit for Polysaccharide-Polyphenol Plants (DP441). The RNA was then reverse-transcribed into cDNA using the HiScript III 1st Strand cDNA Synthesis Kit (Nanjing Vazyme Biotech Co., Ltd., Nanjing, China) (+gDNA wiper). Primers for RT-qPCR were designed using Primer3Plus (https://www.primer3plus.com (accessed on 10 September 2024)) to target eight highly expressed *BpHSF* genes (*BpHSFB2a*, *BpHSFB2b*, *BpHSFA2a*, *BpHSFA5*, *BpHSFB1b*, *BpHSFA9*, *BpHSFA2b*, and *BpHSFC1a*), with all the primer sequences listed in Table S6. 18S rRNA and α-Tubulin were used as internal reference genes [78]. The RT-qPCR reaction mixture consisted of 7 µL ddH_2_O, 1 µL cDNA, 2 µL specific primers, and 10 µL SYBR Premix Ex Taq™ II (TaKaRa BIO Inc., Beijing, China). The RT-qPCR cycling conditions were as follows: pre-denaturation at 95 °C for 30 s, followed by denaturation at 95 °C for 5 s, annealing at 60 °C for 30 s, amplification at 95°C for 5 s, annealing at 60 °C for 60 s, and a melting curve analysis (95 °C for 5 s, 60 °C for 60 s, and 50°C for 30 s). The relative expression levels of *BpHSF* genes were calculated using the 2^−ΔΔCT^ method [79]. Statistical analyses were performed using one-way ANOVA followed by Duncan’s multiple comparison test in SPSS 22.0 software. Graphical representation of data was carried out using GraphPad Prism 10.0.

## 5. Conclusions

In this study, 21 *BpHSF* genes were identified in *Betula platyphylla*, which were classified into three distinct subfamilies. A comprehensive analysis of these genes and their encoded proteins, encompassing phylogenetic relationships, conserved motifs, gene structure, and secondary and tertiary structures, revealed their highly conserved nature. Chromosomal localization and synteny analyses further provided valuable insights into the evolutionary relationships of the *BpHSF* gene family. Tissue-specific expression profiling indicated that these genes may have specialized roles in the growth and development of *B. platyphylla*. In particular, *BpHSFA2a* showed significant upregulation in response to high-temperature stress. These findings lead us to propose that *BpHSFA2a* acts as a key regulatory gene, playing a crucial role in enhancing heat tolerance by modulating the plant’s heat stress response mechanisms. Consequently, *BpHSFA2a* emerges as a promising candidate for improving the heat stress resilience of *B. platyphylla*.

## Figures and Tables

**Table 1:** Physicochemical properties of proteins encoded by the 21 *BpHSF* genes in *B. platyphylla.*

Number	Gene Name	MF	MW	pI	II	AI	GRAVY	SL
1	*BpHSFA1a*	C_2707_H_4259_N_761_O_880_S_27_	62,410.79	4.85	67.51	68.25	−0.615	Nucleus
2	*BpHSFA1b*	C_2443_H_3836_N_696_O_789_S_23_	56,318.90	5.14	52.68	69.32	−0.643	Nucleus
3	*BpHSFA2a*	C_1760_H_2779_N_509_O_536_S_14_	40,094.36	5.94	63.46	64.21	−0.908	Nucleus
4	*BpHSFA2b*	C_1878_H_2982_N_540_O_608_S_13_	43,270.37	4.86	58.74	79.59	−0.517	Nucleus
5	*BpHSFA3*	C_2831_H_4389_N_759_O_901_S_18_	64,050.62	4.77	57.57	70.76	−0.511	Nucleus
6	*BpHSFA4*	C_1993_H_3072_N_552_O_615_S_16_	45,118.61	5.50	39.18	67.27	−0.476	Nucleus
7	*BpHSFA5*	C_2363_H_3686_N_678_O_765_S_13_	54,250.12	5.50	59.56	63.02	−0.774	Nucleus
8	*BpHSFA6*	C_1673_H_2617_N_473_O_507_S_12_	37,853.77	5.39	56.14	78.14	−0.700	Nucleus
9	*BpHSFA9*	C_2463_H_3909_N_681_O_822_S_22_	56,918.52	4.73	70.13	69.69	−0.707	Nucleus
10	*BpHSFA8a*	C_1022_H_1586_N_284_O_309_S_7_	23,019.97	6.15	43.09	69.59	−0.763	Nucleus
11	*BpHSFA8b*	C_1795_H_2828_N_490_O_578_S_23_	41,258.52	4.80	49.06	67.60	−0.772	Nucleus
12	*BpHSFB1a*	C_1046_H_1643_N_291_O_315_S_11_	23,687.97	8.78	49.70	66.80	−0.726	Nucleus
13	*BpHSFB1b*	C_1055_H_1653_N_305_O_364_S_9_	24,722.10	4.90	30.82	56.64	−0.707	Nucleus
14	*BpHSFB2a*	C_1475_H_2318_N_416_O_468_S_10_	33,687.74	5.47	53.72	67.22	−0.687	Nucleus
15	*BpHSFB2b*	C_1579_H_2492_N_454_O_528_S_6_	36,476.24	4.64	54.29	74.85	−0.702	Nucleus
16	*BpHSFB3*	C_1013_H_1615_N_293_O_316_S_10_	23,275.34	8.61	58.36	64.75	−0.766	Nucleus
17	*BpHSFB4a*	C_1583_H_2405_N_441_O_469_S_10_	35,438.78	7.31	57.09	64.90	−0.708	Nucleus
18	*BpHSFB4b*	C_1718_H_2657_N_475_O_508_S_13_	38,510.65	8.20	60.45	74.93	−0.462	Nucleus
19	*BpHSFC1a*	C_1602_H_2508_N_446_O_496_S_19_	36,561.37	5.29	68.69	66.41	−0.550	Nucleus
20	*BpHSFC1b*	C_1139_H_1770_N_310_O_349_S_15_	25,871.35	5.76	54.05	67.00	−0.340	Nucleus
21	*BpHSFC1c*	C_822_H_1226_N_216_O_267_S_9_	18,694.60	4.34	55.65	53.63	−0.351	Nucleus

## Data Availability

Data are contained within the article and the Supplementary Materials.

## Supplementary Materials

The following supporting information can be downloaded at: https://www.mdpi.com/article/10.3390/ijms26010172/s1.
