# Supplementary material for: Genome-Wide Characterization of the Heat Shock Transcription Factor Gene Family in Betula platyphylla Reveals Promising Candidates for Heat Tolerance
Source: Int J Mol Sci. 2024 Dec 28;26(1):172. doi: 10.3390/ijms26010172 (PMC11720272; doi:10.3390/ijms26010172)
Supplement: Supplementary file 1 [file ijms-26-00172-s001.zip › Supplementary Figure and table/Supplementary Figure S1.pdf]

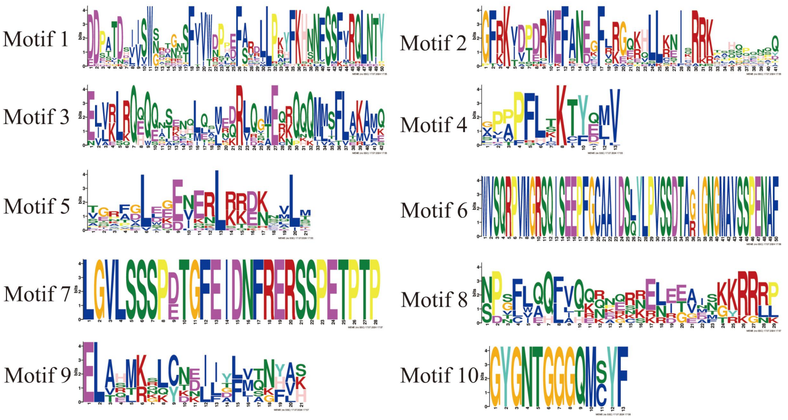

Supplementary Figure S1 Letter stacks at each location show the amino acid sequences of different conserved motifs
