# Supplementary material for: Genome-Wide Characterization of the Heat Shock Transcription Factor Gene Family in Betula platyphylla Reveals Promising Candidates for Heat Tolerance
Source: Int J Mol Sci. 2024 Dec 28;26(1):172. doi: 10.3390/ijms26010172 (PMC11720272; doi:10.3390/ijms26010172)
Supplement: Supplementary file 1 [file ijms-26-00172-s001.zip › Supplementary Figure and table/Supplementary Figure S2.pdf]

Supplementary Figure S2 Prediction of secondary structural properties of BpHsf proteins

Protein	Proportion of secondary structure element (%)				Distribution of secendary structure element
	α-helix	Extended strand	β-turn	Random coil	
BpHSFA1a	32.33	8.66	3.89	55.12	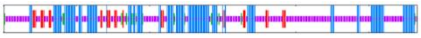
BpHSFA1b	32.67	8.37	4.78	54.18	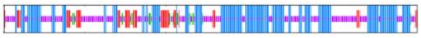
BpHSFA2a	51.86	6.02	5.44	36.68	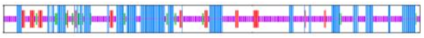
BpHSFA2b	56.40	5.18	4.27	34.15	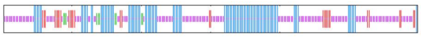
BpHSFA3	50.13	6.46	5.43	37.98	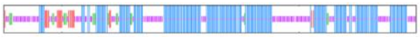
BpHSFA4	28.25	9.19	3.47	59.10	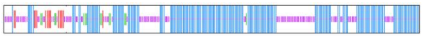
BpHSFA5	33.50	15.27	5.91	45.32	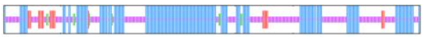
BpHSFA6	36.23	6.42	1.86	55.49	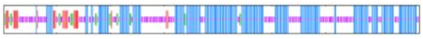
BpHSFA8a	50.51	11.22	7.14	31.12	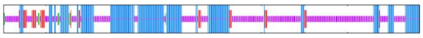
BpHSFA8b	46.52	6.13	2.79	44.57	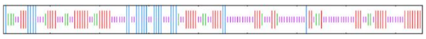
BpHSFA9	42.83	11.00	4.32	41.85	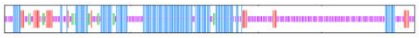
BpHSFB1a	49.03	4.85	7.77	38.35	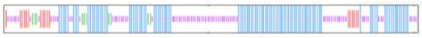
BpHSFB1b	41.05	13.10	4.37	41.48	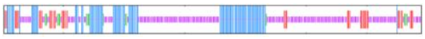
BpHSFB2a	32.78	9.70	3.34	54.18	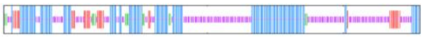
BpHSFB2b	36.28	7.93	3.05	52.74	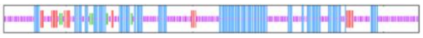
BpHSFB2c	46.53	9.41	4.95	39.11	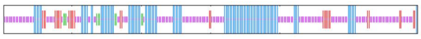
BpHSFB4a	35.95	9.80	4.90	49.35	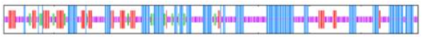
BpHSFB4b	25.51	11.30	4.35	58.84	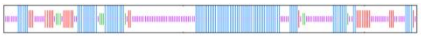
BpHSFC1a	34.98	8.05	4.64	53.32	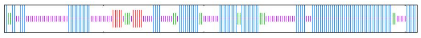
BpHSFC1b	25.32	23.18	10.73	40.77	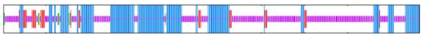
BpHSFC1c	13.45	29.82	9.94	46.78	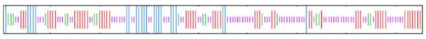
